# 18β-Glycyrrhetinic Acid Protects against Cholestatic Liver Injury in Bile Duct-Ligated Rats

**DOI:** 10.3390/antiox11050961

**Published:** 2022-05-12

**Authors:** Pin-Ho Pan, Ya-Yu Wang, Shih-Yi Lin, Su-Lan Liao, Yu-Fang Chen, Wei-Chi Huang, Chun-Jung Chen, Wen-Ying Chen

**Affiliations:** 1 Department of Veterinary Medicine, National Chung Hsing University, Taichung City 402, Taiwan; pph.pgi@gmail.com (P.-H.P.); vh950420@gmail.com (W.-C.H.); 2 Department of Pediatrics, Tungs’ Taichung MetroHarbor Hospital, Taichung City 435, Taiwan; 3 Department of Family Medicine, Taichung Veterans General Hospital, Taichung City 407, Taiwan; yywang@vghtc.gov.tw; 4 Center for Geriatrics and Gerontology, Taichung Veterans General Hospital, Taichung City 407, Taiwan; sylin@vghtc.gov.tw; 5 Institute of Clinical Medicine, National Yang Ming Chiao Tung University, Taipei City 112, Taiwan; 6 Department of Medical Research, Taichung Veterans General Hospital, Taichung City 407, Taiwan; slliao@vghtc.gov.tw; 7 Department of Medical Laboratory Science, I-Shou University, Kaohsiung City 840, Taiwan; chentina830@gmail.com; 8 Department of Medical Laboratory Science and Biotechnology, China Medical University, Taichung City 404, Taiwan

**Keywords:** cholestasis, FXR, hepatoprotection

## Abstract

18β-Glycyrrhetinic acid is a nutraceutical agent with promising hepatoprotective effects. Its protective mechanisms against cholestatic liver injury were further investigated in a rodent model of extrahepatic cholestasis caused by Bile Duct Ligation (BDL) in rats. The daily oral administration of 18β-Glycyrrhetinic acid improved liver histology, serum biochemicals, ductular reaction, oxidative stress, inflammation, apoptosis, impaired autophagy, and fibrosis. 18β-Glycyrrhetinic acid alleviated the BDL-induced hepatic and systemic retention of bile acids, matrix-producing cell activation, hepatic collagen deposition, Transforming Growth Factor beta-1/Smad activation, malondialdehyde elevation, glutathione reduction, High Mobility Group Box-1/Toll-Like Receptor-4 activation, NF-κB activation, inflammatory cell infiltration/accumulation, Interleukin-1β expression, Signal Transducer and Activator of Transcription-1 activation, Endoplasmic Reticulum stress, impairment autophagy, and caspase 3 activation. Conversely, the protein expression of Sirt1, Farnesoid X Receptor, nuclear NF-E2-Related Factor-2, Transcription Factor EB, bile acid efflux transporters, and LC3-II, as well as the protein phosphorylation of AMP-Activated Protein Kinase, was promoted in 18β-Glycyrrhetinic acid-treated BDL rats. The hepatoprotective effects of 18β-Glycyrrhetinic acid in the present investigation correlated well with co-activation and possible interactions among Sirt, FXR, and Nrf2. The concurrent or concomitant activation of Sirt1, FXR, and Nrf2 not only restored the homeostatic regulation of bile acid metabolism, but also alleviated oxidative stress, inflammation, apoptosis, impaired autophagy, and fibrosis.

## 1. Introduction

Cholestasis is a hepatobiliary disorder with a heterogeneous etiology. Primary biliary cholangitis and primary sclerosing cholangitis are clinically reported cholestatic liver diseases. Unfortunately, the effectiveness of therapeutic drugs for the treatment of hepatic cholestasis is still limited. Currently, the more commonly therapeutic approaches for treating primary biliary cholangitis are secondary bile acid ursodeoxycholic acid or Farnesoid X Receptor (FXR) agonist obeticholic acid. However, a relatively small number of patients show any beneficial response [1,2]. Therefore, a better understanding of the molecular mechanisms underlying the pathogenesis of cholestasis is of paramount importance as it may aid in the development of a therapeutic strategy and could help to identify a drug candidate capable of combating hepatic cholestasis.

Cholestatic liver diseases are characterized by a functional impairment of bile formation and/or bile flow resulting from defective secretion by hepatocytes or cholangiocytes, or the mechanical obstruction of bile flow at sites of intrahepatic or extrahepatic bile ducts. Besides genetic disorders, cholestasis may arise from a number of hepatic complications, including drug or xenobiotic toxicity, pathogen infection, hepatobiliary malignancies, obstruction of the biliary tract, and even a complication of liver transplantation [3,4,5,6]. Independent of etiologies, cholestasis is highlighted by the abnormal intrahepatic and systemic accumulation of bile acids, with accompanying toxic responses in liver parenchymal cells, oxidative stress, and inflammation, predisposing and progressing to fibrosis, cirrhosis, and even end-stage liver disease [7,8]. Normally, bile acids act as an important metabolic integrator. Once dysregulation occurs, hydrophobic bile acids stimulate cholangiocyte proliferation and activation, and induce free radical generation. The cholestasis-accompanied pathological retention of bile acids has diverse and progressive effects on hepatocytes, hepatic stellate cells, and Kupffer cells, leading to oxidative stress, hepatic inflammation, fibrosis, and programmed hepatic cell death. Parallel studies reveal that the improvement of bile flow and alleviation of free radical generation, inflammation, and programmed cell death confer beneficial effects on cholestatic liver injury. Experimental studies further indicate the hepatoprotective potential of antioxidants and agents with anti-oxidative ability [9,10,11,12,13]. The aforementioned phenomena highlight the substantial roles of accumulated bile acids, oxidative stress, inflammation, and programmed cell death in the pathogenesis of cholestatic liver injury and underscore their importance in interventions for the therapeutic treatment of cholestasis.

Sirtuin 1 (Sirt1), a conserved NAD^+^-dependent class III histone deacetylase, has a central role in coordinating a wide range of metabolic processes by deacetylating and activating the transcription and activity of metabolic regulators and signaling integrators [14]. Sirt1 has been implicated in the positive regulation of bile acid metabolism and autophagy, as well as the negative regulation of oxidative stress, inflammation, and cell death by acting on FXR, NF-E2-Related Factor-2 (Nrf2), Transcription Factor EB (TFEB), AMP-Activated Protein Kinase (AMPK), and High Mobility Group Box 1 (HMGB1) [15,16,17,18]. Dysregulated Sirt1 and parallel changes in impaired FXR and Nrf2, oxidative stress, and upregulated HMGB1 have been demonstrated in cholestatic liver injury and their reversal in hepatoprotective interventions [11,12,18,19,20,21,22]. Thus, Sirt1 and Sirt1-related molecules are targets for the investigation of cholestatic pathogenesis and the exploration of treatment candidates.

Traditional Chinese medicine and nutraceutical agents have been reported to have hepatoprotective effects, and several active ingredients have been identified, including *Glycyrrhiza glabra* L. (licorice). A large number of biological compounds have been isolated from the roots of licorice, and the main constituent is glycyrrhizin, accounting for about 10% of the licorice root dry weight. Glycyrrhizin is a glycoside of glycyrrhetinic acid with two residues of glucuronic acid. After oral administration, glycyrrhizin is rapidly and near completely metabolized to glycyrrhetinic acid by intestinal bacteria. Glycyrrhetinic acid, particularly, 18β-Glycyrrhetinic acid, is the main active metabolite of glycyrrhizin and is responsible for most pharmacological properties. Studies have demonstrated the pharmacological and health-promoting effects of 18β-Glycyrrhetinic acid, including antioxidant, anti-inflammation, anticancer, and metabolic regulation [23,24,25]. Additionally, 18β-Glycyrrhetinic shows promising hepatoprotective properties in numerous types of liver diseases, including ischemia/reperfusion-, acetaminophen-, viral infection-, cyclophosphamide-, methotrexate-, free fatty acid-, pyrrolizidine alkaloid-, and carbon tetrachloride-induced liver injury [26,27,28,29,30,31,32,33,34,35]. Regarding cholestasis, 18β-Glycyrrhetinic acid protects against α-Naphthylisothiocyanate-induced intrahepatic cholestasis, and its beneficial effect is blocked by Sirt1 inhibitor [6,36]. The delivery of M6P-HSA-glycyrrhetinic acid to hepatic stellate cells attenuates hepatic fibrogenesis in a rodent study [37]. In vitro, 18β-Glycyrrhetinic acid protects rat hepatocytes against bile acid toxicity [38]. Although evidence-based data are limited, licorice and licorice derivatives have been prescribed in the treatment of infant biliary atresia [31,39,40]. The findings imply a potentially active role of 18β-Glycyrrhetinic acid centered on bile acid-oriented liver injury. Though 18β-Glycyrrhetinic acid’s hepatoprotective potential attracts attention, currently, no experimental evidence exists on its effectiveness against extrahepatic cholestatic liver injury.

α-Naphthylisothiocyanate, a hepatic toxin, is metabolized by hepatocytes and the resultant metabolites secret to bile, thus inducing toxic effects on cholangiocytes. Thus, α-Naphthylisothiocyanate feeding in rodents provides an experimental model for the study of intrahepatic cholestasis [6,36]. To simulate extrahepatic liver injury, experimental models are frequently established in rodents by producing common Bile Duct Ligation (BDL) and scission. Portal hypertension and bile acid retention are early signs of the obstructive cholestasis and the changes can progress to liver injury, fibrosis, cirrhosis, and even failure [41]. Previously, we investigated pathophysiological alterations in extrahepatic cholestatic liver injury caused by BDL in Sprague Dawley rats and found that certain dietary nutraceutical supplements had hepatoprotective effects [41,42,43,44,45]. To extend the scope of 18β-Glycyrrhetinic acid studies and gain insights into its extrahepatic cholestatic hepatoprotection, the present study aimed to ascertain the hepatoprotective properties of 18β-Glycyrrhetinic acid in a rat model of extrahepatic cholestasis with a view to clarifying the underlying molecular basis of its actions. Based on the aforementioned studies, we hypothesize that Sirt1 and Sirt1-related molecules have substantial roles in 18β-Glycyrrhetinic acid-mediated hepatoprotection.

## 2. Materials and Methods

### 2.1. Animals and Surgical Operation

The Taichung Veterans General Hospital Institutional Animal Care and Use Committee reviewed and approved the protocols of experimental procedures (IACUC approval code: La-1101778; IACUC approval date: 18 February 2021). Adult male Sprague Dawley rats (200–250 g), purchased from BioLASCO (Taipei, Taiwan), were housed in a controlled animal room under a standard 12 h light/12 h dark cycle with food and water *ad libitum*. The test study consisted of five groups: sham, BDL, and BDL receiving one of three doses of 18β-Glycyrrhetinic acid (10, 30, and 50 mg/kg) (*n* = 4 per group). There were four groups (two sham groups and two BDL groups) in the treatment study, and one dose of 18β-Glycyrrhetinic acid (50 mg/kg) was delivered (*n* = 8 per group). BDL and sham operation in rats were performed, as described in our previously reported papers, under anesthesia with isoflurane. For sham operations, all steps were the same, except for common Bile Duct Ligation [43,44,45]. Saline and 18β-Glycyrrhetinic acid were administered by oral gavage once daily at 9–10 AM for 4 weeks. One week after dietary supplementation, rats were subjected to sham and BDL operation, respectively. At the end of the treatment course, rats were euthanized. Blood samples were withdrawn from the femoral artery, and the liver tissues were quickly removed and stored in a deep freezer until analyses or were paraffin-embedded.

### 2.2. Biochemical Analysis

Serum levels of Aspartate Aminotransferase (AST), Alanine Aminotransferase (ALT), Alkaline Phosphatase (ALP), total bilirubin, total cholesterol, triglycerides, and γ-Glutamyl Transpeptidase (γ-GT) were measured using automated standardized procedures (Roche Hitachi 917/747, Mannheim, Germany). The serum and hepatic contents of total bile acids were measured using a commercially available total bile acids assay kit (Diazyme Laboratories, Poway, CA, USA). The serum and hepatic contents of Interleukin-1β (IL-1β) were measured using an Enzyme-Linked Immunosorbent Assay (ELISA) (R&D Systems, Minneapolis, MN, USA).

### 2.3. Histological and Immunohistochemical Examination

The paraffin-embedded liver tissues were cut to 5 μm thickness for subsequent histological and immunohistochemical examination [43,44,45]. Histological detection was carried out on deparaffinized sections by staining with Hematoxylin/Eosin (H&E) and Sirius Red. Immunohistochemistry was performed by incubating the tissue sections with α-Smooth Muscle Actin (1:100, α-SMA, Calbiochem Biotechnology, San Diego, CA, USA) and Cluster of Differentiation 68 (1:100, CD68, Invitrogen, Carlsbad, CA, USA) antibody, followed by Horseradish Peroxidase-labeled IgG (IgG-HRP). The color reactions were developed with diaminobenzidine. The histopathological change in bile duct hyperplasia was graded with an established scoring system in accordance with our previously reported protocols [43,44,45]. The definition of the one- to five-point scoring was as follows: 1 = minimal (<1%); 2 = slight (1–25%); 3 = moderate (26–50%); 4 = moderate/severe (51–75%); 5 = severe/high (76–100%).

### 2.4. RNA Isolation and Quantitative Real-Time Reverse Transcriptase Polymerase Chain Reaction (RT-PCR)

Total RNAs of liver tissues were extracted with TRIzol RNA Isolation Reagents (ThermoFisher Scientific, Waltham, MA, USA). cDNA synthesis and quantitative real-time PCR were performed on ABI StepOne^TM^ (Applied Biosystems, Foster City, CA, USA) using SYBR Green-based detection [43,44,45]. All results were normalized with 18S rRNA expression and calculated using the ΔΔCT method. Oligonucleotides for the amplification were listed as follows: 5′-CCTGACTATCTGAAAGCCATTTGG and 5′-CACAGTGCTGCAATGCTCTACAC for Manganese Superoxide Dismutase (Mn-SOD, NM_017051.2); 5′-GCGGTGAACCAGTTGTGGTG and 5′-AGCCACATTGCCCAGGTCTC for Copper/Zinc-SOD (Cu/Zn-SOD, NM_017050.1); 5′-CATTCGAACGTCTGCCCTAT and 5′-GTTTCTCAGGCTCCCTCTCC for 18S rRNA (NC_051336.1).

### 2.5. Tissue Preparation and Western Blot

Total cellular proteins were extracted from the dissected liver tissues with tissue protein extraction reagents (T-PER^TM^, ThermoFisher Scientific, Waltham, MA, USA) containing 1% protease inhibitor cocktail. For Western blotting, the obtained protein extracts were separated using SDS-PAGE, transferred to a PVDF membrane, blocked with 5% skim milk, and then incubated with antibodies recognizing: Bile Salt Export Pump (1:1000, BSEP), Multidrug Resistance Associated Protein 3 (1:1000, Mrp3), Mrp4 (1:1000), FXR (1:1000), α-SMA (1:1000), TGF-β1 (1:500), Smad2/3 (1:1000), Signal Transducer and Activator of Transcription 1 (1:1000, *Stat1*), phospho-Stat1 (1:500, P-Stat1, Tyr-701), HMGB1 (1:500), Toll-Like Receptor-4 (1:1000, TLR4), MyD88 (1:1000), Extracellular Signal-Regulated Kinase (1:1000, ERK), phospho-ERK (1:500, P-ERK, Thr-202/Tyr-204), c-Jun N-Terminal Kinase (1:1000, JNK), phospho-JNK (1:500, P-JNK, Thr-183/Tyr-185), p65 (1:1000), phospho-p65 (1:500, P-p65, Ser-536), CD68 (1:1000), Inositol-Requiring Enzyme 1 (1:1000, *IRE1*), TNF Receptor-Associated Factor-2 (1:500, TRAF2), LC3 (1:1000), Beclin1 (1:500), Nrf2 (1:500), Sirt1 (1:500), TFEB (1:1000), p62/SQSTM1 (1:1000), AMPK (1:1000), phospho-AMPK (1:500, P-AMPK), Glyceraldehyde-3-Phosphate Dehydrogenase (1:3000, GAPDH) (Santa Cruz Biotechnology, Santa Cruz, CA, USA), phospho-Smad2/3 (1:500, P-Smad2/3, Thr-8, ThermoFisher Scientific, Waltham, MA, USA), and phospho-IRE1 (1:500, P-IRE1, Ser-724, Abcam, Cambridge, UK). After reacting with IgG-HRP and incubating with enhanced chemiluminescence Western blotting reagents, the proteins of interest were visualized and quantified using a G:BOX mini multi-fluorescence and chemiluminescence imaging system (Syngene, Frederick, MD, USA).

### 2.6. Collagen Measurement

Total cellular proteins were extracted from the dissected liver tissues with tissue protein extraction reagents (T-PER^TM^, ThermoFisher Scientific, Waltham, MA, USA) containing 1% protease inhibitor cocktail. The hepatic contents of collagen (50 μg protein extracts) were measured using a commercial assay kit (Sigma-Aldrich, St. Louis, MO, USA).

### 2.7. Measurement of Hydroxyproline Content

The dissected liver tissues were subjected to protein hydrolysis and the measurement of hydroxyproline content using a Hydroxyproline Colorimetric Assay Kit (BioVision, Mountain View, CA, USA) according to the manufacturer’s instructions.

### 2.8. Gelatinase Zymography

Total cellular proteins were extracted from the dissected liver tissues with tissue protein extraction reagents (T-PER^TM^, ThermoFisher Scientific, Waltham, MA, USA) containing 1% protease inhibitor cocktail. The obtained protein extracts (50 μg) were separated using 8% SDS-PAGE containing gelatin (0.5 mg/mL). Thereafter, the proteolytic zones in the gels were visualized through staining with Coomassie Brilliant R-250 [43,44,45].

### 2.9. Preparation of Nuclear Extracts and Electrophoretic Mobility Shift Assay (EMSA)

The dissected liver tissues were subjected to nuclear protein extraction and EMSA using an extraction kit (NE-PER Nuclear and Cytoplasmic Extraction Kit, ThermoFisher Scientific, Waltham, MA, USA) and a commercial EMSA assay kit (LightShift^TM^ Chemiluminescent EMSA Kit, ThermoFisher Scientific, Waltham, MA, USA), respectively, according to the manufacturer’s instructions [43,44,45]. Oligonucleotides for the detection were as follows: 5′-AGTTGAGGGGACTTTCCCAGGC for NF-κB. The protein/DNA complexes were visualized and quantified using a G:BOX mini multi-fluorescence and chemiluminescence imaging system (Syngene, Frederick, MD, USA) after incubation with enhanced chemiluminescence Western blotting reagents.

### 2.10. Caspase 3 Activity Assay

The dissected liver tissues were subjected to protein extraction and measurement of caspase 3 activity using a Caspase Fluorometric Assay Kit (BioVision, Mountain View, CA, USA), according to the manufacturer’s instructions.

### 2.11. Measurement of Lipid Peroxidation

The serum and hepatic contents of lipid peroxidation product were measured using a Thiobarbituric Acid Reactive Substances (TBARS) assay kit (ZeptoMetrix, Buffalo, NY, USA). The levels of the lipid peroxidation products were presented as Malondialdehyde (MDA) equivalents.

### 2.12. Measurement of Glutathione (GSH)

The dissected liver tissues were subjected to the measurement of Glutathione (GSH) using a Glutathione Assay Kit (Cayman Chemical, Ann Arbor, MI, USA), according to the manufacturers’ instructions.

### 2.13. NRF2/ARE Luciferase Reporter Assay

A stable HepG2 cell line carrying a pTA-ARE-luciferase reporter vector with 4 repeats of antioxidant response binding sites was purchased from Signosis (Santa Clara, CA, USA) and maintained in Dulbecco’s Modified Eagle’s Medium (DMEM) containing 10% Fetal Bovine Serum (FBS). Sub-confluent cells grown on 6-well plates were treated with various concentrations of 18β-Glycyrrhetinic acid for 24 h. Cells were harvested, lysed, and subjected to the measurement of luciferase activity using Luciferase Reporter Assay Kit (BioVision, Mountain View, CA, USA) according to the manufacturer’s instructions.

### 2.14. Statistical Analysis

Results are expressed as the mean values ± Standard Deviation (SD). All statistical tests were performed using the GraphPad Prism 8.0 Software (2020). Depending on the number of groups and variables, two-way Analysis of Variance (ANOVA) with Bonferroni or Dunnett post hoc test was performed. A value of *p* < 0.05 was considered statistically significant.

## 3. Results

### 3.1. 18β-Glycyrrhetinic Acid Alleviated BDL-Induced Liver Injury and Ductular Reaction

**Figure 1:**
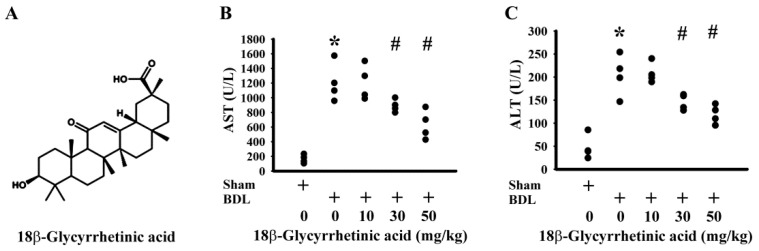
18β-Glycyrrhetinic acid decreased serum biochemical parameters in the test study. Chemical structure of 18β-Glycyrrhetinic acid is shown (**A**). Serum levels of AST (**B**) and ALT (**C**) activity were measured. * *p* < 0.05 vs. the sham vehicle group and # *p* < 0.05 vs. the BDL vehicle group, *n* = 4.

**Figure 2:**
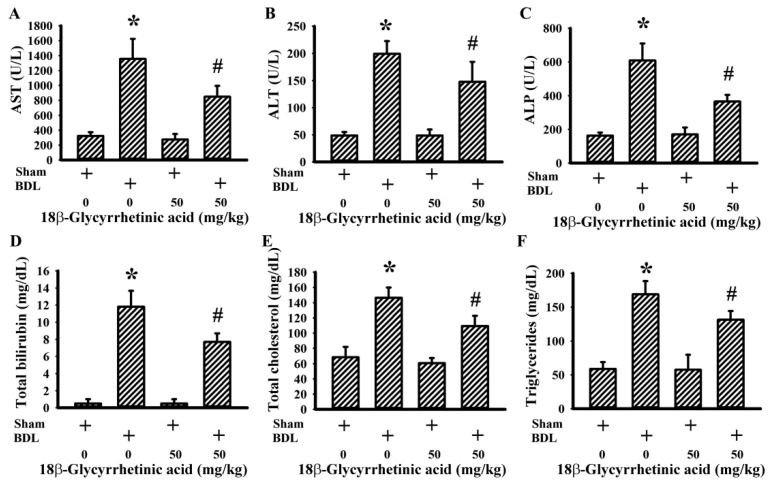
18β-Glycyrrhetinic acid decreased serum biochemical parameters in the treatment study. Serum levels of AST activity (**A**), ALT activity (**B**), ALP activity (**C**), total bilirubin (**D**), total cholesterol (**E**), and triglycerides (**F**) were measured. * *p* < 0.05 vs. the sham vehicle group and # *p* < 0.05 vs. the BDL vehicle group, *n* = 8.

**Figure 3:**
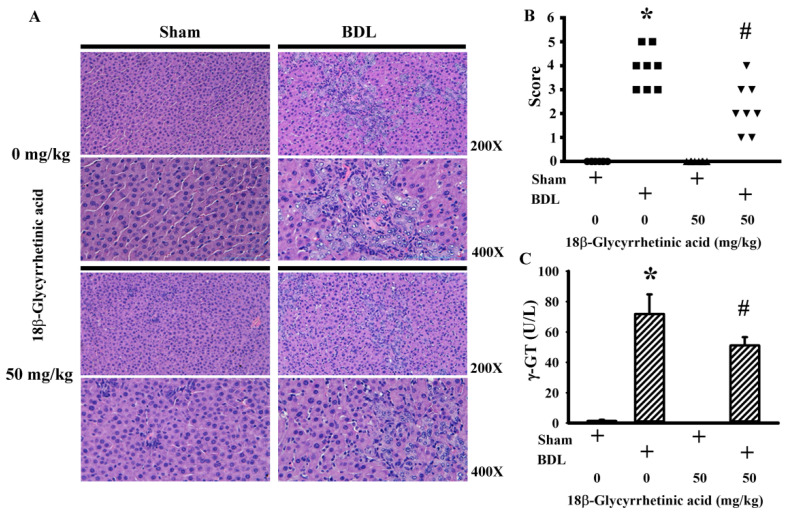
18β-Glycyrrhetinic acid alleviated BDL-induced liver histopathological changes in the treatment study. The paraffin-embedded liver tissues were subjected to histological examination. Representative photomicrographs of H&E staining are shown. Original magnification is 200× and 400× (**A**). The severity of bile duct hyperplasia is scored (**B**). Serum levels of γ-GT activity were measured (**C**). * *p* < 0.05 vs. the sham vehicle group and # *p* < 0.05 vs. the BDL vehicle group, *n* = 8.

Hepatoprotective doses of 18β-Glycyrrhetinic acid (Figure 1A) were first evaluated in the test study. Over the course of the study, there was a reduction in body mass and average food intake in BDL rats when compared with sham rats, and these decreases were not remarkably changed by daily supplementation with various doses of 18β-Glycyrrhetinic acid (data not shown). Based on the serum levels of AST (Figure 1B) and ALT (Figure 1C), 18β-Glycyrrhetinic acid at doses of 10, 30, and 50 mg/kg displayed hepatoprotective effects, particularly at the two higher doses. Thereafter, a dose of 50 mg/kg 18β-Glycyrrhetinic acid was supplemented in the following treatment study. BDL rats showed elevated serum levels of AST (Figure 2A), ALT (Figure 2B), ALP (Figure 2C), total bilirubin (Figure 2D), total cholesterol (Figure 2E), and triglycerides (Figure 2F); hepatic histopathological changes (Figure 3A); and increased scores of bile duct hyperplasia (Figure 3B) and serum γ-GT activity (Figure 3C). Daily supplementation with 18β-Glycyrrhetinic acid improved BDL-accompanied changes in serum biochemical and liver histopathology (Figure 2 and Figure 3). The findings suggest that 18β-Glycyrrhetinic acid supplementation alleviated BDL-induced liver injury and ductular reaction.

### 3.2. 18β-Glycyrrhetinic Acid Alleviated BDL-Induced Impaired Bile Acid Metabolism

**Figure 4:**
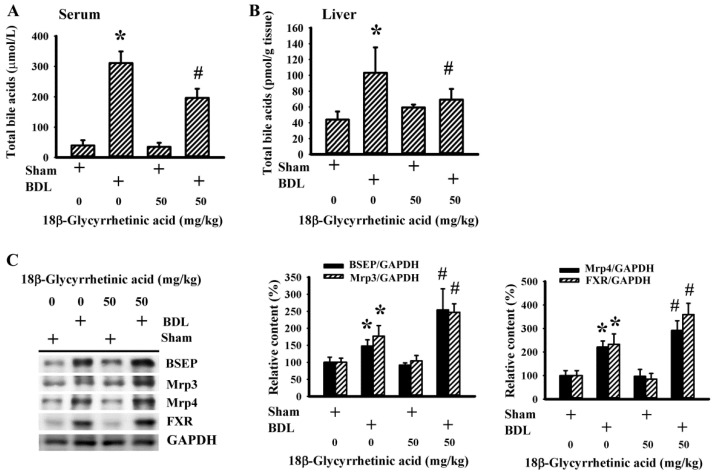
18β-Glycyrrhetinic acid alleviated BDL-induced retention of bile acids in the treatment study. The serum (**A**) and hepatic levels (**B**) of total bile acids were measured. (**C**) Protein contents in liver tissues were measured using Western blot with indicated antibodies. Representative blots and the quantitative data are shown. * *p* < 0.05 vs. the sham vehicle group and # *p* < 0.05 vs. the BDL vehicle group, *n* = 8.

Cholestasis is typically characterized by abnormal intrahepatic and systemic retention of bile acids [9]. BDL rats showed elevated serum (Figure 4A) and hepatic (Figure 4B) contents of total bile acids, and the increments decreased following treatment with 18β-Glycyrrhetinic acid. The elevated retention of bile acids in BDL rats paralleled the slightly increased protein expression of metabolic nuclear receptor FXR and hepatic bile acid efflux transporters, including BSEP, Mrp3, and Mrp4. Importantly, their expressions were further upregulated upon daily supplementation with 18β-Glycyrrhetinic acid (Figure 4C). The findings indicate an improvement in bile acid metabolism and transport induced by 18β-Glycyrrhetinic acid in BDL rats.

### 3.3. 18β-Glycyrrhetinic Acid Alleviated BDL-Induced Hepatic Collagen Deposition

**Figure 5:**
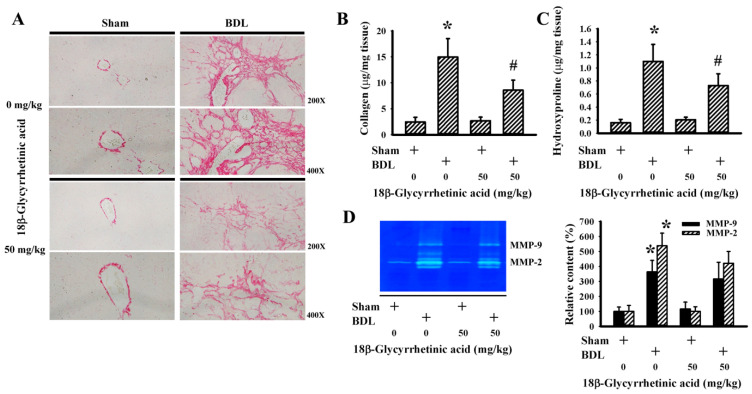
18β-Glycyrrhetinic acid alleviated BDL-induced hepatic collagen deposition in treatment study. (**A**) The paraffin-embedded liver tissues were subjected to Sirius Red staining. Representative photomicrographs are shown. Original magnification is 200× and 400×. (**B**) Collagen contents in liver tissues were measured. (**C**) Hydroxyproline contents in liver tissues were measured. (**D**) MMP-2 and MMP-9 activities of proteins isolated from liver tissues were measured using zymographic assay. Representative zymographs and the quantitative data are shown. * *p* < 0.05 vs. the sham vehicle group and # *p* < 0.05 vs. the BDL vehicle group, *n* = 8.

The hepatic accumulation of extracellular matrix proteins in parenchyma is a sign of a histopathological change in cholestasis [46]. Results of histological examination via Sirius Red staining (Figure 5A) and the biochemical measurement of hepatic contents of collagen (Figure 5B) and hydroxyproline (Figure 5C) revealed an elevated deposition of hepatic collagen in BDL rats and an alleviative effect of 18β-Glycyrrhetinic acid. Although hepatic Matrix Metalloproteinase-2 (MMP-2) and MMP-9 activities were elevated in BDL rats, 18β-Glycyrrhetinic acid had little effect on the increased proteolytic activities (Figure 5D). These findings suggest that 18β-Glycyrrhetinic acid supplementation improved BDL-induced hepatic collagen deposition.

### 3.4. 18β-Glycyrrhetinic Acid Alleviated BDL-Induced Myofibroblast Activation

**Figure 6:**
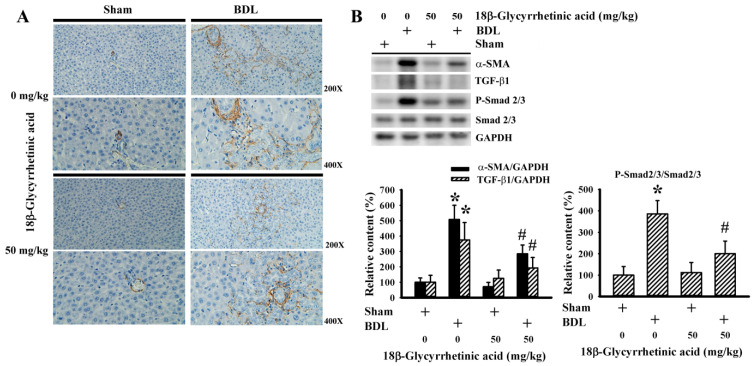
18β-Glycyrrhetinic acid alleviated BDL-induced fibrotic signaling in the treatment study. (**A**) The paraffin-embedded liver tissues were subjected to immunohistochemical examination with anti-α-SMA antibody. Representative photomicrographs of α-SMA immunoreactivity are shown. Original magnification is 200× and 400×. (**B**) Protein contents in liver tissues were measured using Western blot with indicated antibodies. Representative blots and the quantitative data are shown. * *p* < 0.05 vs. the sham vehicle group and # *p* < 0.05 vs. the BDL vehicle group, *n* = 8.

TGF-β1/Smad signaling is a critical fibrogenic driver, acting on portal myofibroblasts and hepatic stellate cells, which are α-SMA immunoreactive and determinant matrix-producing cells [46]. BDL rats showed the increased immunohistochemical detection of hepatic α-SMA immunoreactivity (Figure 6A) and protein content (Figure 6B), and these elevations were reduced by 18β-Glycyrrhetinic acid. Together with the alteration of α-SMA immunoreactivity and protein content, parallel changes were found in the hepatic protein content of TGF-β1 and the protein phosphorylation of Smad2/3 (Figure 6B). The findings suggest that the alleviative effects of 18β-Glycyrrhetinic acid were associated with a reduction in hepatic TGF-β1/Smad signaling and matrix-producing cells.

### 3.5. 18β-Glycyrrhetinic Acid Alleviated BDL-Induced Hepatic Inflammation

**Figure 7:**
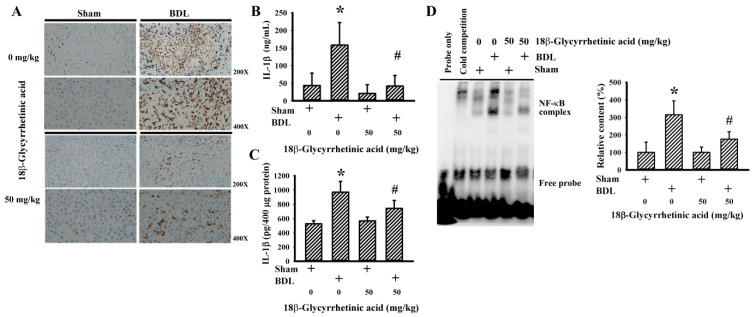
18β-Glycyrrhetinic acid alleviated BDL-induced inflammatory NF-κB in the treatment study. (**A**) The paraffin-embedded liver tissues were subjected to immunohistochemical examination with anti-CD68 antibody. Representative photomicrographs of CD68 immunoreactivity are shown. Original magnification is 200× and 400×. The serum samples (**B**) and liver tissues (**C**) were subjected to ELISA for the measurement of IL-1β. (**D**) NF-κB DNA binding activity of nuclear proteins isolated from liver tissues was measured using EMSA. Cold competition assay was conducted using nuclear proteins obtained from the BDL vehicle group. Representative blots and the quantitative data are shown. * *p* < 0.05 vs. the sham vehicle group and # *p* < 0.05 vs. the BDL vehicle group, *n* = 8.

**Figure 8:**
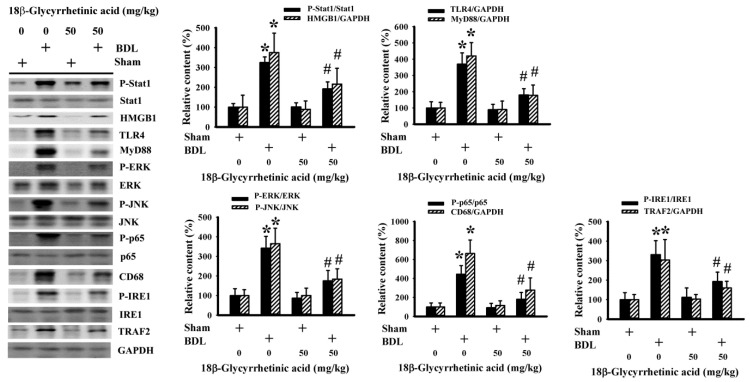
18β-Glycyrrhetinic acid alleviated BDL-induced inflammatory signaling in the treatment study. Protein contents in liver tissues were measured using Western blot with indicated antibodies. Representative blots and the quantitative data are shown. * *p* < 0.05 vs. the sham vehicle group and # *p* < 0.05 vs. the BDL vehicle group, *n* = 8.

Transcription factor NF-κB governs a transcriptional program in coordinating inflammatory cell activation and cytokine expression, leading to the progression of cholestatic liver injury [43,44,45]. The results of the hepatic immunohistochemical detection of CD68 immunoreactivity (Figure 7A), hepatic protein content of CD68 (Figure 8), serum level of IL-1β (Figure 7B), hepatic level of IL-1β (Figure 7C), and hepatic NF-κB activity (Figure 7D) showed the hepatic activation of NF-κB-guided monocytes/macrophages and cytokines in BDL rats. Parallel activation was found in several intracellular signaling molecules that are important to the NF-κB-related inflammatory axis, including hepatic content of HMGB1, TLR4, MyD88, and TRAF2 protein, as well as Stat1, ERK, JNK, p65, and IRE1 protein phosphorylation (Figure 8). BDL-induced hepatic inflammatory responses were alleviated by 18β-Glycyrrhetinic acid (Figure 7 and Figure 8). The findings suggest that 18β-Glycyrrhetinic acid can alleviate BDL-induced hepatic inflammation involving the alleviation of Stat1-, HMGB1/TLR4-, Mitogen-Activated Protein Kinases (MAPKs)-, Endoplasmic Reticulum (ER) stress-, and NF-κB-related machinery.

### 3.6. 18β-Glycyrrhetinic Acid Alleviated BDL-Induced Oxidative Stress

**Figure 9:**
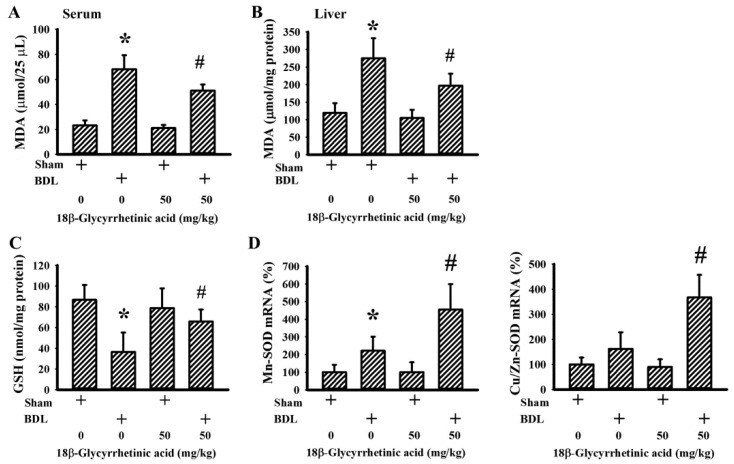
18β-Glycyrrhetinic acid alleviated BDL-induced oxidative stress in the treatment study. The serum (**A**) and hepatic levels (**B**) of MDA were measured using the TBARS assay. (**C**) Hepatic contents of GSH were measured. (**D**) Total RNAs were extracted from the obtained liver tissues and subjected to the measurement of Mn-SOD and Cu/Zn-SOD mRNA. * *p* < 0.05 vs. the sham vehicle group and # *p* < 0.05 vs. the BDL vehicle group, *n* = 8.

Free radicals and the consequences of oxidative stress have been implicated in the pathogenesis of cholestatic liver injury by inducing oxidative damage, hepatic inflammation, and fibrosis. Antioxidants are reported to have substantial effects against cholestatic liver injury [47]. Elevated serum (Figure 9A) and hepatic (Figure 9B) contents of lipid peroxidation product MDA, along with the reduced hepatic content of GSH (Figure 9C), were found in BDL rats. The elevation of MDA and reduction in GSH in BDL rats were alleviated by 18β-Glycyrrhetinic acid (Figure 9A–C). BDL rats showed marginal elevation in the hepatic mRNA level of Mn-SOD and Cu/Zn-SOD, while the levels were further increased by 18β-Glycyrrhetinic acid (Figure 9D); that is, 18β-Glycyrrhetinic acid is shown to be capable of alleviating BDL-induced oxidative stress.

### 3.7. 18β-Glycyrrhetinic Acid Improved BDL-Altered Hepatic Signaling

**Figure 10:**
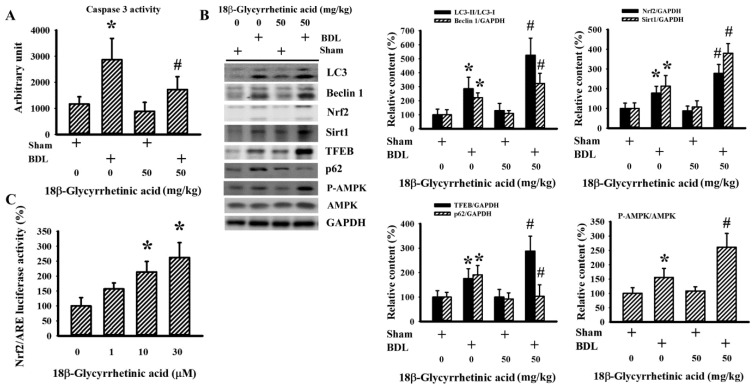
18β-Glycyrrhetinic acid improved BDL-altered hepatic signaling molecules in the treatment study. (**A**) Liver tissues were collected and subjected to the measurement of caspase 3 activity. (**B**) Protein contents in liver tissues were measured using Western blot with indicated antibodies. Representative blots and the quantitative data are shown. * *p* < 0.05 vs. the sham vehicle group and # *p* < 0.05 vs. the BDL vehicle group, *n* = 8. (**C**) NRF2/ARE luciferase reporter stable HepG2 cells were treated with various concentrations of 18β-Glycyrrhetinic acid for 24 h. Cells were harvested, lysed, and subjected to the measurement of luciferase activity. * *p* < 0.05 vs. untreated control, *n* = 4.

To further demonstrate the hepatoprotective effects of 18β-Glycyrrhetinic acid, hepatic changes in the upstream regulators of oxidative stress, inflammation, and cell death were determined. The results of caspase 3 activity (Figure 10A), LC3-II generation, elevated Beclin1, and accumulated p62 (Figure 10B) revealed the occurrence of hepatic apoptosis and impaired autophagy in BDL rats. 18β-Glycyrrhetinic acid alleviated the changes, except for the generation of LC3-II and Beclin1, which actually increased (Figure 10A,B). Increased Nrf2, Sirt1, TFEB protein content, and AMPK protein phosphorylation (Figure 10B) in the hepatic tissues of BDL rats were found. The increases were further augmented by 18β-Glycyrrhetinic acid (Figure 10B). The effects of 18β-Glycyrrhetinic acid on Nrf2 expression were alternatively demonstrated by in vitro reporter assay. 18β-Glycyrrhetinic acid stimulated luciferase activity in HepG2 cells carrying the NRF2/ARE luciferase reporter vector (Figure 10C). These findings suggest that 18β-Glycyrrhetinic acid was able to decrease BDL-induced hepatic apoptosis and impaired autophagy, probably involving the activation of Nrf2, Sirt1, TFEB, and AMPK.

## 4. Discussion

The present study revealed a hepatoprotective effect of 18β-Glycyrrhetinic acid on extrahepatic cholestatic liver injury and shed light on its mechanistic actions, which appear to be correlated with Sirt1 signaling. In extrahepatic cholestasis, BDL rats developed the hepatic and systemic retention of bile acids accompanied by serum and histological parameters of liver injury, oxidative stress, inflammation, fibrosis, apoptosis, and impaired autophagy. Daily supplementation with 18β-Glycyrrhetinic acid alleviated BDL-accompanied changes in BDL rats. At the molecular level, the enhanced protein expression of metabolic regulators, such as Sirt1, FXR, Nrf2, and TFEB, as well as the protein phosphorylation of AMPK, was demonstrated in 18β-Glycyrrhetinic acid-supplemented BDL rats. In contrast to other liver diseases, the impairment of bile flow and the consequences of the hepatic retention of bile acids are the primary and dominant causes of intrahepatic and extrahepatic cholestasis development [3,4,5,6]. 18β-Glycyrrhetinic acid has diverse pharmacological properties with benefits against a number of disorders, including liver diseases [23,24,25,26,27,28,29,30,31,32,33,34,35,36]. However, the utility and effectiveness of 18β-Glycyrrhetinic acid against extrahepatic cholestatic liver injury remained unexplored. There are previous studies showing the ameliorative effects of 18β-Glycyrrhetinic acid on distinct types of liver injury through diverse modes of action, including Sirt1, FXR, Nrf2, HMGB1, TLR4, TGF-β1, cytochrome P450, and PPAR-γ [26,27,28,29,30,31,32,33,34,35,36]. Additionally, the current study further revealed the potential involvement of Stat1, TFEB, AMPK, and ER stress in the hepatoprotection of 18β-Glycyrrhetinic acid. Despite a lack of direct evidence, the current findings demonstrated a hepatoprotective effect of 18β-Glycyrrhetinic acid against cholestatic liver injury, probably through the actions of Sirt1 signaling. However, the conclusion should be further confirmed by direct approaches

Extracts of licorice roots, glycyrrhizin, and 18β-Glycyrrhetinic acid have been reported to increase Sirt1 expression or activity in in vivo and in vitro studies [6,48,49,50]. Among the upstream regulators, Sirt1 expression and activity are downregulated by the actions of TGF-β1/Smad and toxic bile acids, and are promoted by AMPK [21,48,51,52]. Additionally, Sirt1 is able to enhance AMPK activity and inhibit TGF-β1 signaling [53,54]. The preservation of hepatic Sirt1 expression in BDL rats by 18β-Glycyrrhetinic acid was paralleled by the decreased TGF-β1/Smad signaling and hepatic content of bile acids, along with increased AMPK phosphorylation. 18β-Glycyrrhetinic acid attenuates TGF-β1/Smad signaling in hepatic stellate cells [55]. Currently, its effect on AMPK signaling is not clear. Our findings suggest that increased TGF-β1/Smad signaling and hepatic bile acids could be the cause of dysregulated Sirt1 in the hepatic tissues of BDL rats. Therefore, the axes of TGF-β1/Smad, bile acids, and AMPK could be action targets of 18β-Glycyrrhetinic acid in regulating Sirt1 actions, and the fine-tuning of the balance among Sirt1, TGF-β1/Smad, bile acids, and AMPK is critical to the biological activity of Sirt1.

Clinically, a dysregulated Sirt1 level and its activity have been found in the livers of patients with extrahepatic cholestasis and nonalcoholic fatty liver disease [21,56]. A parallel Sirt1 dysregulation has also been reported in a wide range of rodent models of liver diseases, including α-Naphthylisothiocyanate, thioacetamide, and carbon tetrachloride [6,52,57]. The overexpression of Sirt1 and its activators has shown benefits and has been implicated in the beneficial treatments against liver injury, including cholestatic liver injury [6,22,57,58,59,60,61]. In in vitro hepatocytes, Sirt1 displays a direct protective effect against bile acid toxicity [21]. Despite its impairment in the aforementioned studies, its upregulation has also been found in diseased livers [62]. Regardless of its upregulation or downregulation in diseased livers, all studies indicate that Sirt1 possesses hepatoprotective properties. Thus, Sirt1 represents a candidate with pharmacological value and is a possible underlying mediator in the hepatic actions of 18β-Glycyrrhetinic acid.

Bile acid is a pathogenic molecule critical to the initiation and progression of cholestatic liver injury [52]. Normally, bile flow is dynamically regulated at multiple steps, including bile acid synthesis, canalicular secretion, intestinal absorption, and basolateral efflux. FXR, a nuclear receptor and transcription factor, is a master sensor of intracellular bile acid level and has a fundamental role in bile acid metabolism. Upon engagement with endogenous ligand bile acid, FXR restores bile homeostasis by repressing CYP7A1-mediated cholesterol catabolism and bile acid synthesis, as well as Sodium Taurocholate Cotransporting Polypeptide (NTCP)-mediated bile acid absorption. Moreover, it induces the expression of phase II drug metabolism enzymes and bile acid efflux transporters, such as BSEP, Mrp3, and Mrp4 [63]. Hepatic FXR is a proposed mediator responsible for Sirt1-directed metabolic effects because Sirt1 is a critical transcriptional and transactivational regulator of FXR [18]. The BDL-accompanied impairment of bile acid metabolism and the consequences of the retention of bile acids were improved by 18β-Glycyrrhetinic acid. The restoration or enhancement of the Sirt1 and hepatic expression of FXR, BSEP, Mrp3, and Mrp4, along with a reduction in the total cholesterol level, was demonstrated in 18β-Glycyrrhetinic acid-treated BDL rats. Taken together, the Sirt1-governed repression of bile acid synthesis and the promotion of bile acid efflux represent candidate modes of action in resolving the hepatic and systemic burden of bile acids in BDL rats after daily supplementation with 18β-Glycyrrhetinic acid.

The nutrient and metabolic roles of bile acids are completed by rounds of physiological conjugation, secretion, biotransformation, and enterohepatic circulation. These dynamic processes increase bile acid solubility, reduce bile acid cytotoxic potential, and convert them to biologically active metabolites. Once interrupted, hepatic accumulated bile acids are of high hydrophobicity and acquire the ability to cause mitochondrial dysfunction, free radical generation, inflammation, and hepatic cell death, leading to fibrosis and cholestatic liver injury [7,8,52]. Besides their role as the cells’ energy powerhouse, mitochondria exert effects on oxidative stress, inflammation, and cell death [47]. Bile acids have direct toxic effects on isolated mitochondria [64]. In cholestasis patients, cholestatic liver injury is accompanied by mitochondrial dysfunction and mitochondrial DNA damage [65]. Mitochondrial dysfunction-associated oxidative stress, inflammation, fibrosis, and cell death have been implicated in rodent models of cholestatic liver injury. Importantly, mitochondria-targeted antioxidants and the inhibition of mitochondrial fission have alleviative effects against hepatic oxidative stress, inflammation, stellate cell activation, cell death, and fibrosis [47,66,67]. These beneficial effects against cholestatic liver injury can also be achieved by increased Nrf2 expression and its activators [11,68]. Enhanced hepatic Nrf2 activation has been observed in primary biliary cirrhosis patients with ursodeoxycholic acid treatment and has a role in the therapeutic response to ursodeoxycholic acid [69]. Nrf2 has positive effects on bile acid homeostasis and is able to increase the expression of BSEP, Mrp3, and Mrp4 in the hepatic tissues of cholestatic mice. Its genetic deletion in mice causes the development of cholestatic phenotypes [68,70]. Sirt1 activators alleviate cholestatic liver injury through the activation of FXR and Nrf2 [22]. Since mutual activation between FXR and Nrf2 has been reported [71,72], our findings suggest active roles of FXR and Nrf2 linking Sirt1 and bile acid metabolism, as well as their functional interplay in mediating the hepatoprotective actions of 18β-Glycyrrhetinic acid against cholestatic liver injury.

Transcription factor Nrf2, well-known for its antioxidant property in combating oxidative stress through the Nrf2/Antioxidant Response Element (ARE) pathway, still displays multiple biological effects besides bile acid metabolism. Nrf2 alleviates mitochondrial dysfunction [73] and limits hepatic stellate cell activation through the inhibition of the TGF-β1/Smad pathway [74]. The inhibition of HMGB1/TLR4, MAPKs, NF-κB, AP-1, and NLRP3 inflammasome signaling has been demonstrated in the Nrf2-mediated suppression of inflammation [75,76,77,78]. Regarding programmed cell death, Nrf2 promotes autophagy, but alleviates apoptosis. It is noteworthy that Nrf2 has been identified as a novel autophagy inducer through the TFEB-controlled lysosomal biogenesis [79]. In 18β-Glycyrrhetinic acid-treated BDL rats, increased hepatic Nrf2 and TFEB levels paralleled reductions in MDA contents, TGF-β1/Smad signaling, myofibroblast activation, fibrosis, HMGB1/TLR4 signaling, NF-κB signaling, IL-1β expression, inflammatory cell activation and infiltration, caspase 3 activity, and impaired autophagy, as well as increases in GSH contents, Beclin1 expression, and LC3-II generation. In addition to the homeostatic regulation of bile acid metabolism, the current findings imply that the adaptive and cytoprotective actions of Nrf2 represent alternative mechanisms of protection against cholestatic liver injury caused by 18β-Glycyrrhetinic acid involving the suppression of oxidative stress, inflammation, programmed cell death, impaired autophagy, and fibrosis.

While the increased or decreased expression of Sirt1, FXR, and Nrf2 has been demonstrated in diseased livers, their corresponding overexpression or activators show hepatoprotective effects against cholestatic liver injury [1,11,12,18,20,21,22]. 18β-Glycyrrhetinic acid is one of the most studied active ingredients of licorice, with promising hepatoprotective activity, and has been shown to have a positive effect on the expression and activity of Sirt1, FXR, and Nrf2 [6,25,28,29]. Our in vitro reporter assay study provided evidence indicating a promoting effect of 18β-Glycyrrhetinic acid on Nrf2 expression and action. Although the detailed regulatory mechanisms of Sirt1, FXR, and Nrf2 expression and activity in 18β-Glycyrrhetinic acid-treated BDL rats remain to be fully elucidated, the experimental findings of the current study suggest their roles in the restoration of bile acid metabolism and alleviation of cholestatic liver injury. It is reasonable to expect that the beneficial actions of Sirt1, FXR, and Nrf2 may extend to 18β-Glycyrrhetinic acid broad-spectrum hepatoprotective activity against various types of liver injury. However, further in-depth investigation is urgently needed to achieve a better understanding of 18β-Glycyrrhetinic acid-mediated hepatoprotection.

Studies indicate that the Stat1 and ER stress represent alternative regulators in coordinating TLR4 and MAPKs inflammatory responses and are also components of liver injury [18,80,81,82]. 18β-Glycyrrhetinic acid-protected hepatic inflammation and liver injury in BDL rats paralleled a reduction in Stat1 protein phosphorylation, IRE1 protein phosphorylation, and TRAF2 protein expression, implying the involvement of Stat1 signaling and ER stress. Therefore, ER stress and Stat1 signaling could be alternative targets of 18β-Glycyrrhetinic acid in combating cholestatic liver injury.

Despite current hepatoprotective findings, there are limitations regarding the mechanistic actions of 18β-Glycyrrhetinic acid. First, 18β-Glycyrrhetinic acid in the absence of BDL had a limited effect on the assayed parameters. Insufficient tissue concentrations of 18β-Glycyrrhetinic acid could be one cause of this. Additionally, the existing perturbance of metabolic processes in BDL could be a prerequisite for the actions of 18β-Glycyrrhetinic acid. Second, although current findings revealed an association between the Sirt1 signaling and accompanied beneficial effects in 18β-Glycyrrhetinic acid-treated BDL rats, there was a lack of direct evidence in supporting their functional correlation. Except Sirt1, ER stress, Stat1 signaling, cytochrome P450 enzyme, and bile acids represent alternative targets having roles in the therapeutic benefits of 18β-Glycyrrhetinic acid. Finally, despite the positive effects of 18β-Glycyrrhetinic acid revealed in this study, chronic licorice consumption is associated with adverse effects, including hypertension, hypokalemia, and other signs of mineralocorticoid excess [83]. Therefore, individuals with existing cardiovascular diseases undergoing licorice or 18β-Glycyrrhetinic acid supplementation should be studied. The aforementioned limitations slightly weaken the scientific value and translational implication of the current findings. Thus, a deeper investigation is needed.

## 5. Conclusions

The hepatic and systemic retention of bile acids are cytotoxic determinants in cholestatic liver injury caused by extrahepatic BDL. Mechanistically, the hepatoprotective effects of 18β-Glycyrrhetinic acid in the present investigation could be well correlated with co-activation and possible interactions among Sirt, FXR, and Nrf2. The concurrent or concomitant activation of Sirt1, FXR, and Nrf2 not only restores the homeostatic regulation of bile acid metabolism, but also alleviates oxidative stress, inflammation, programmed cell death, impaired autophagy, and fibrosis, involving the interplay of multiple intracellular signaling pathways. The promising effects and pleiotropic targets achieved by 18β-Glycyrrhetinic acid warrant further investigation to explore its advanced utility and application.

## Data Availability

All of the data is contained within the article.
